# A community effort to optimize sequence-based deep learning models of gene regulation

**DOI:** 10.1038/s41587-024-02414-w

**Published:** 2024-10-11

**Authors:** Abdul Muntakim Rafi, Daria Nogina, Dmitry Penzar, Dohoon Lee, Danyeong Lee, Nayeon Kim, Sangyeup Kim, Dohyeon Kim, Yeojin Shin, Il-Youp Kwak, Georgy Meshcheryakov, Andrey Lando, Arsenii Zinkevich, Byeong-Chan Kim, Juhyun Lee, Taein Kang, Eeshit Dhaval Vaishnav, Payman Yadollahpour, Random Promoter DREAM Challenge Consortium, Susanne Bornelöv, Fredrik Svensson, Maria-Anna Trapotsi, Duc Tran, Tin Nguyen, Xinming Tu, Wuwei Zhang, Wei Qiu, Rohan Ghotra, Yiyang Yu, Ethan Labelson, Aayush Prakash, Ashwin Narayanan, Peter Koo, Xiaoting Chen, David T. Jones, Michele Tinti, Yuanfang Guan, Maolin Ding, Ken Chen, Yuedong Yang, Ke Ding, Gunjan Dixit, Jiayu Wen, Zhihan Zhou, Pratik Dutta, Rekha Sathian, Pallavi Surana, Yanrong Ji, Han Liu, Ramana V. Davuluri, Yu Hiratsuka, Mao Takatsu, Tsai-Min Chen, Chih-Han Huang, Hsuan-Kai Wang, Edward S. C. Shih, Sz-Hau Chen, Chih-Hsun Wu, Jhih-Yu Chen, Kuei-Lin Huang, Ibrahim Alsaggaf, Patrick Greaves, Carl Barton, Cen Wan, Nicholas Abad, Cindy Körner, Lars Feuerbach, Benedikt Brors, Yichao Li, Sebastian Röner, Pyaree Mohan Dash, Max Schubach, Onuralp Soylemez, Andreas Møller, Gabija Kavaliauskaite, Jesper Madsen, Zhixiu Lu, Owen Queen, Ashley Babjac, Scott Emrich, Konstantinos Kardamiliotis, Konstantinos Kyriakidis, Andigoni Malousi, Ashok Palaniappan, Krishnakant Gupta, Prasanna Kumar S, Jake Bradford, Dimitri Perrin, Robert Salomone, Carl Schmitz, Chen JiaXing, Wang JingZhe, Yang AiWei, Sun Kim, Jake Albrecht, Aviv Regev, Wuming Gong, Ivan V. Kulakovskiy, Pablo Meyer, Carl G. de Boer

**Affiliations:** 1 University of British Columbia, Vancouver, British Columbia Canada; 2 Faculty of Bioengineering and Bioinformatics, Lomonosov Moscow State University, Moscow, Russia; 3 Vavilov Institute of General Genetics, Russian Academy of Sciences, Moscow, Russia; 4 AIRI, Moscow, Russia; 5 Institute of Protein Research, Russian Academy of Sciences, Pushchino, Russia; 6 Seoul National University, Seoul, South Korea; 7 Chung-Ang University, Seoul, South Korea; 8 Yandex, Moscow, Russia; 9 Broad Institute of MIT and Harvard, Cambridge, MA USA; 10 Sequome, Inc., South San Francisco, CA USA; 11 Sage Bionetworks, Seattle, WA USA; 12 Genentech, San Francisco, CA USA; 13 University of Minnesota, Minneapolis, MN USA; 14 Health Care and Life Sciences, IBM Research, New York, NY USA; 15 University of Cambridge, Cambridge, UK; 16 University College London, London, UK; 17 University of Nevada, Reno, Reno, NV USA; 18 University of Washington, Seattle, WA USA; 19 Syosset High School, Syosset, NY USA; 20 Cold Spring Harbor Laboratory, Cold Spring Harbor, NY USA; 21 Friends Academy, Locust Valley, NY USA; 22 Half Hollow Hills High School, Dix Hills, NY USA; 23 Jericho High School, Jericho, NY USA; 24 Cincinnati Children’s Hospital Medical Center, Cincinnati, OH USA; 25 The Wellcome Centre for Anti-Infectives Research, Dundee University, Dundee, UK; 26 University of Michigan, Ann Arbor, MI USA; 27 Sun Yat-sen University, Guangzhou, China; 28 Australian National University, Canberra, Australian Capital Territory Australia; 29 Northwestern University, Evanston, IL USA; 30 Stony Brook University, Stony Brook, New York, NY USA; 31 Niigata University School of Medicine, Niigata, Japan; 32 Graduate Program of Data Science, National Taiwan University and Academia Sinica, Taipei, Taiwan; 33 Research Center for Information Technology Innovation, Academia Sinica, Taipei, Taiwan; 34 ANIWARE, Taipei, Taiwan; 35 Molecular Sciences and Digital Innovation Center, GGA Corp, Taipei, Taiwan; 36 Institute of Biomedical Sciences, Academia Sinica, Taipei, Taiwan; 37 Development Center for Biotechnology, Taipei, Taiwan; 38 Interdisciplinary Artificial Intelligence Center, National Chengchi University, Taipei, Taiwan; 39 Graduate Institute of Biomedical Electronics and Bioinformatics, National Taiwan University, Taipei, Taiwan; 40 School of Medicine, China Medical University, Taichung, Taiwan; 41 Birkbeck, University of London, London, UK; 42 German Cancer Research Institute (DKFZ), Heidelberg, Germany; 43 Faculty of Engineering Sciences, Heidelberg University, Heidelberg, Germany; 44 St. Jude Children’s Research Hospital, Memphis, TN USA; 45 Berlin Institute of Health at Charité, Universitätsmedizin Berlin, Berlin, Germany; 46 Global Blood Therapeutics, South San Francisco, CA USA; 47 University of Southern Denmark, Odense, Denmark; 48 University of Tennessee at Knoxville, Knoxville, TN USA; 49 Aristotle University of Thessaloniki, Thessaloniki, Greece; 50 School of Chemical and Biotechnology, SASTRA Deemed University, Thanjavur, India; 51 National Centre for Cell Science (NCCS), Pune, India; 52 Queensland University of Technology, Brisbane, Queensland Australia; 53 Beijing Normal University–Hong Kong Baptist University United International College, Zhuhai, China

**Keywords:** Gene regulation

## Abstract

A systematic evaluation of how model architectures and training strategies impact genomics model performance is needed. To address this gap, we held a DREAM Challenge where competitors trained models on a dataset of millions of random promoter DNA sequences and corresponding expression levels, experimentally determined in yeast. For a robust evaluation of the models, we designed a comprehensive suite of benchmarks encompassing various sequence types. All top-performing models used neural networks but diverged in architectures and training strategies. To dissect how architectural and training choices impact performance, we developed the Prix Fixe framework to divide models into modular building blocks. We tested all possible combinations for the top three models, further improving their performance. The DREAM Challenge models not only achieved state-of-the-art results on our comprehensive yeast dataset but also consistently surpassed existing benchmarks on *Drosophila* and human genomic datasets, demonstrating the progress that can be driven by gold-standard genomics datasets.

## Abstract (web summary)

A benchmarking competition improves tools that predict how regulatory regions control gene expression.

## Main

In eukaryotes, transcription factors (TFs) have a crucial role in regulating gene expression and are critical components of the *cis*-regulatory mechanism^1–6^. TFs compete with nucleosomes and each other for DNA binding and can enhance each other’s binding through biochemical cooperativity and mutual competition with nucleosomes^7–10^. While the field has made substantial progress in characterizing regulatory mechanisms^11–19^, a quantitative understanding of *cis*-regulation remains a major challenge. Neural networks (NNs) have shown immense potential in modeling and predicting gene regulation. While different network architectures, such as convolutional NNs (CNNs)^11,12,14,19,20^, recurrent NNs (RNNs)^21^ and transformers^15,17,18,22^, have been used to create genomics models, there is limited research on how NN architectures and training strategies affect their performance for genomics applications. Standard datasets provide a common benchmark to evaluate and compare algorithms, leading to improved performance and continued progress in the field^23^. For instance, the computer vision and natural language processing (NLP) fields have seen an ongoing improvement of NNs facilitated by gold-standard datasets, such as the ImageNet data^23^ and MS COCO^24^. In contrast, because genomics models are often created ad hoc for analyzing a specific dataset, it often remains unclear whether a model’s improved performance results from improved model architecture or better training data. In many cases, the models created are not directly comparable to previous models because of substantial differences in the underlying data used to train and test them.

To address the lack of standardized evaluation and continual improvement of genomics models, we organized the Random Promoter DREAM Challenge^25^. Here, we asked the participants to design sequence-to-expression models and train them on expression measurements of promoters with random DNA sequences. The models would receive a regulatory DNA sequence as input and use it to predict the corresponding gene expression value. We designed a separate set of sequences to test the limits of the models and provide insight into model performance. The top-performing solutions in the challenge exceeded performance of all previous state-of-the-art models for similar data. Our evaluation across various benchmarks revealed that, for some sequence types, model performances approached the previously estimated inter-replicate experimental reproducibility for this datatype^13^, while considerable improvement remains necessary for others. The top-performing models included features inspired by the nature of the experiment and state-of-the-art models from computer vision and NLP, while incorporating training strategies that are better suited to genomics sequence data. To determine how individual design choices affect performance, we created a Prix Fixe framework that enabled modular testing of individual model components, revealing further performance gains. Finally, we benchmarked the top-performing DREAM models on *Drosophila* and human datasets, including predicting expression and open chromatin from DNA sequence, where they consistently surpassed existing state-of-the-art model performances. Recognizing the potential of these models to further the field, we are making all DREAM Challenge models available in an accessible format.

## Results

### The Random Promoter DREAM Challenge and dataset

To generate the competition training data, we conducted a high-throughput experiment to measure the regulatory effect of millions of random DNA sequences (Methods). Prior research has shown that random DNA can display activity levels akin to genomic regulatory DNA because of the incidental occurrence of numerous TF-binding sites (TFBSs)^13,22,26^. Here, we cloned 80-bp random DNA sequences into a promoter-like context upstream of a yellow fluorescent protein (YFP), transformed the resulting library into yeast, grew the yeast in Chardonnay grape must and measured expression by fluorescence-activated cell sorting (FACS) and sequencing^13,27,28^ (Methods). This resulted in a training dataset of 6,739,258 random promoter sequences and their corresponding mean expression values.

We provided these data to the competitors, who could use them to train their model, with two key restrictions. First, competitors were not allowed to use external datasets in any form to ensure that all models are trained on the same dataset. Second, ensemble predictions were also disallowed as they would almost certainly provide a boost in performance but without providing any insight into the best model types and training strategies.

We evaluated the models on a set of ‘test’ sequences designed to probe the predictive ability of the models in different ways. The measured expression levels driven by these sequences were quantified in the same way as the training data but in a separate experiment with more cells sorted per sequence (~100), yielding more accurate estimated expression levels compared to the training data measurements and providing higher confidence in the challenge evaluation. The test set consisted of 71,103 sequences from several promoter sequence types. We included both random sequences and sequences from the yeast genome to get an estimate of performance difference between the random sequences in the training domain and naturally evolved sequences. We also included sequences designed to capture known limitations of previous models trained on similar data, namely sequences at the high-expression and low-expression extremes and sequences designed to maximize the disagreement between the predictions of a previously developed CNN and a physics-informed NN (‘biochemical model’)^13,22^. We previously found that predicting changes in expression between closely related sequences (that is, nearly identical DNA sequences) is substantially more challenging; hence, we included subsets where models had to predict changes that result from single-nucleotide variants (SNVs), perturbations of specific TFBSs and tiling of TFBSs across background sequences^13,22^. Each test subset was given a different weight when scoring the submissions, proportional to the number of sequences in the set and how important we considered it to be (Table 1). For instance, predicting the effects of SNVs on gene expression is a critical challenge for the field because of its relevance to complex trait genetics^29^. Accordingly, a substantial number of SNV sequence pairs were included in the test set and SNVs were given the highest weight. Within each sequence subset, we determined model performance using Pearson’s *r*^2^ and Spearman’s *ρ*, which captured the linear correlation and monotonic relationship between the predicted and measured expression levels (or expression differences), respectively. The weighted sum of each performance metric across test subsets yielded our two final performance measurements, which we called the Pearson score and Spearman score.

**Table 1:** Summary of the test subsets

Subset	No. of sequences	Weight in evaluation metric	Description
All sequences	71,103	1	All sequences in the test data
High	968	0.3	Sequences designed to have high expression
Low	997	0.3	Sequences designed to have low expression
Native	997	0.3	Sequences that are present in the yeast genome
Random	6349	0.3	Random DNA sequences
Challenging	1,953	0.5	Sequences designed to maximize the differences between a convolutional model and a biochemical model trained on the same data
SNVs	44,340 pairs	1.25	Two sequences that differed by only a single base
Motif perturbation (Reb1 + Hsf1)	3,287 pairs	0.3	Two sequences that differed because of perturbations to specific known TFBS
Motif tiling	2,624 pairs	0.4	Two sequences that differed because of tiling known TFBSs across random sequences

**Fig. 1:**
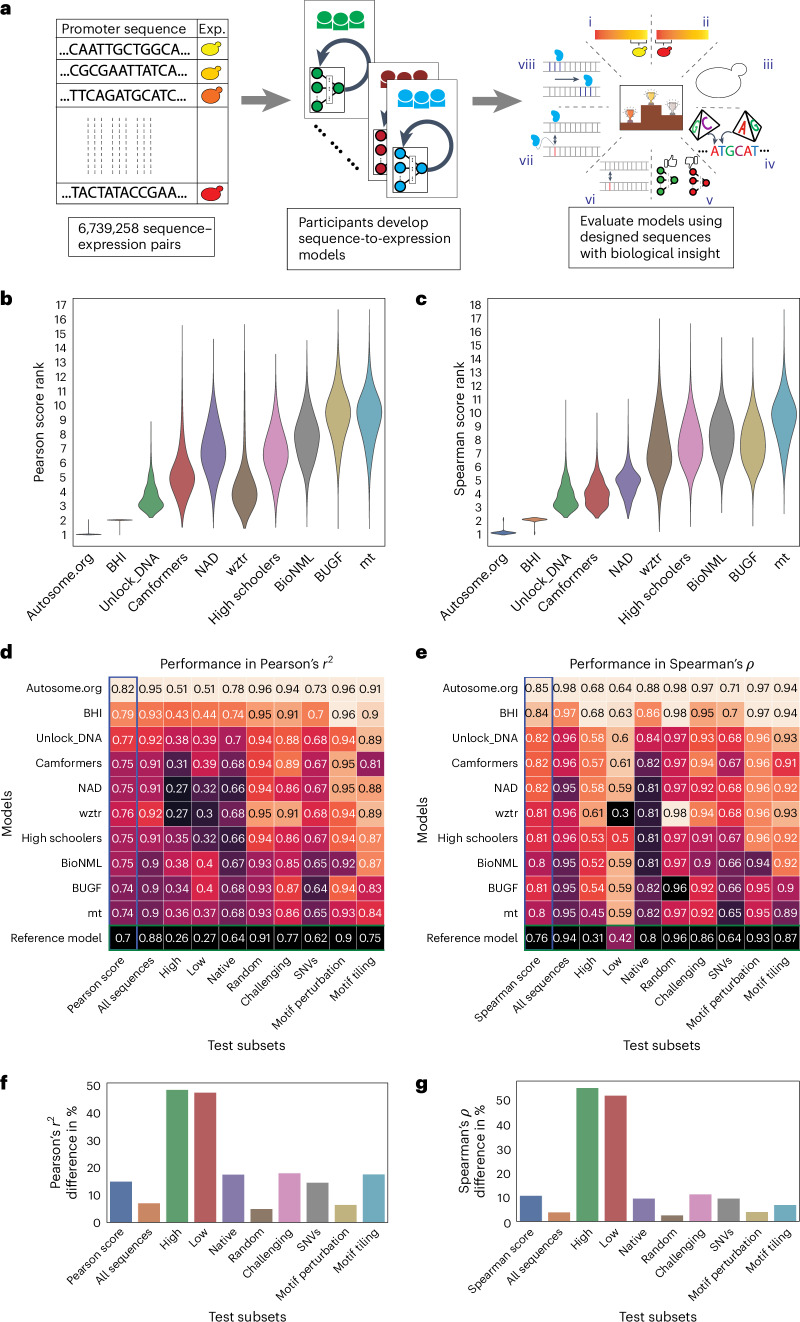
Overview of the challenge. **a**, Left, competitors received a training dataset of random promoters and corresponding expression values. Middle, they continually refined their models and competed for dominance in a public leaderboard. Right, at the end of the challenge, they submitted a final model for evaluation using a test dataset consisting of eight sequence types: (i) high expression, (ii) low expression, (iii) native, (iv) random, (v) challenging, (vi) SNVs, (vii) motif perturbation and (viii) motif tiling. **b**,**c**, Bootstrapping provides a robust comparison of the model predictions. Distribution of ranks in *n* = 10,000 samples from the test dataset (*y* axes) for the top-performing teams (*x* axes) Pearson score (**b**) and Spearman score (**c**). **d**,**e**, Performance of the top-performing teams in each test data subset. Model performance (color and numerical values) of each team (*y* axes) in each test subset (*x* axes) for Pearson’s *r*^2^ (**d**) and Spearman’s *ρ* (**e**). Heat map color palettes are min–max-normalized by column. **f**,**g**, Performance disparities observed between the best and worst models (*x* axes) in different test subsets (*y* axes) for Pearson’s *r*^2^ (**f**) and Spearman’s ρ (**g**). The calculation of the percentage difference is relative to the best model performance for each test subset. Source data

**Extended Data Fig. 1:**
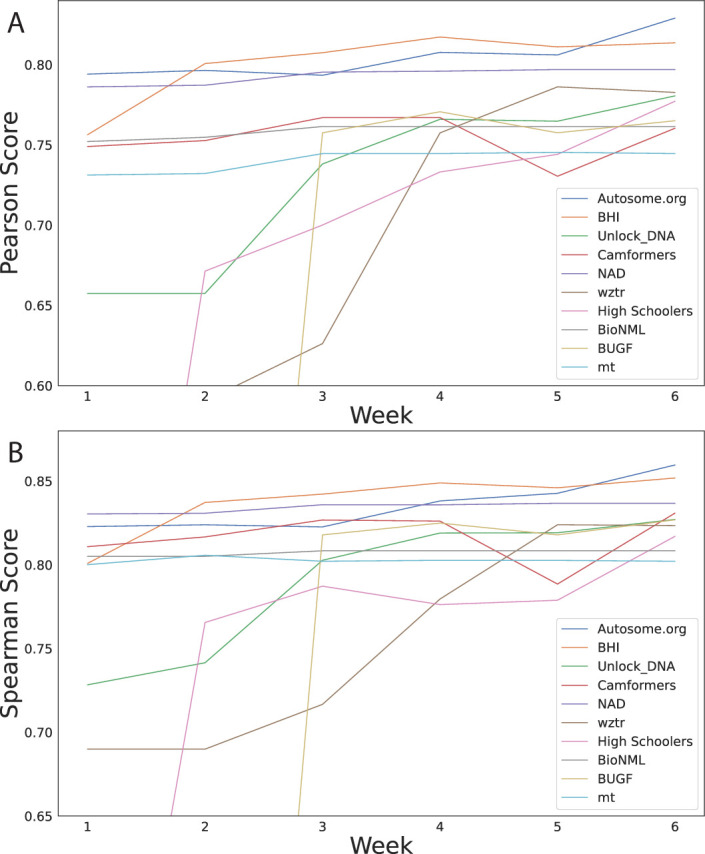
Progression of the top-performing teams’ performance in the DREAM Challenge public leaderboard. **(A,B)** Performance (*y*-axes) in (**A**) Pearson Score and (**B**) Spearman Score achieved by the participating teams (colours) each week (*x*-axes), showcasing the effectiveness of such challenges in motivating the development of better machine learning models. Source data

**Extended Data Fig. 2:**
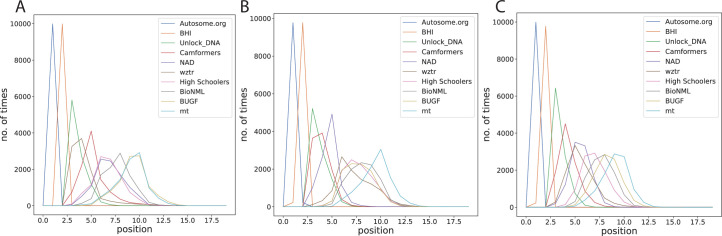
Bootstrapping provides a robust comparison of the model predictions. **(A,B,C)** Frequency (*y*-axes) of ranks (*x*-axes) in (**A**) Pearson Score, (**B**) Spearman Score and combined rank (sum of Pearson Score rank and Spearman Score rank) for $n$=10,000 samples from the test dataset for the top-performing teams. Source data

Our DREAM Challenge ran for 12 weeks in the summer of 2022 and included two evaluation stages: the public leaderboard phase and the private evaluation phase (Fig. 1a). The leaderboard opened 6 weeks into the competition and allowed teams to submit up to 20 predictions on the test data per week. At this stage, we used 13% of the test data for leaderboard evaluation and displayed only the overall Pearson’s *r*^2^, Spearman’s *ρ*, Pearson score and Spearman score to the participants, while keeping the performance on the promoter subsets and the specific sequences used for the evaluation hidden. The participating teams achieved increasing performance each week (Extended Data Fig. 1), showcasing the effectiveness of such challenges in motivating the development of better machine learning models. Over 110 teams across the globe competed in this stage. At the end of the challenge, 28 teams submitted their models for final evaluation. We used the remaining test data (~87%) for the final evaluation (Fig. 1b,c and Extended Data Fig. 2).

### Innovative model designs surpass the state of the art

We retrained the transformer model architecture of Vaishnav et al.^12^, the previous best-performing model for this type of data, on the challenge data and used it as a reference in the leaderboard (‘reference model’). The overall performance of top submissions, all NNs, was substantially better than the reference model. Despite recent prominence of attention-based architectures^22^, only one of the top five submissions in the challenge used transformers, placing third. The best-performing submissions were dominated by fully convolutional NNs, with first, fourth and fifth places taken by them. The best-performing solution was based on the EfficientNetV2 architecture^30,31^ and the fourth and fifth solutions were based on the ResNet architecture^32^. Moreover, all teams used convolutional layers as the starting point in their model design. An RNN with bidirectional long short-term memory (Bi-LSTM) layers^33,34^ placed second. While the teams broadly converged on many similar training strategies (for example, using Adam^35^ or AdamW^36^ optimizers), they also had substantial differences (Table 2).

**Table 2:** Breakdown of the top-performing models into key components

Participant team name	NN architecture type	Input encoding and channels	Input flanking region length	Usage of reverse strand during model training	Train–validation split	Parameters (millions)	Optimizer	Loss function	LR Scheduler	Metric
Autosome.org	CNN (EfficientNetV2)^31^	OHE [6:bases/ NC*/RC*]	70	Data augmentation (additional channel) + model (additional channel)	100:0	1.9	AdamW^36^	Kullback–Leibler divergence	One-cycle LR	*r*, *ρ*^#^
BHI	CNN + RNN (Bi-LSTM)^33^	OHE [4:bases]	30	Post hoc conjoined setting^51^	100:0	6.8	AdamW^36^	Huber	Cosine anneal LR	*r*, *ρ*^#^
Unlock_DNA	Transformer	OHE [6:bases/ N*/M*]	20	Input to model (concatenation with forward strand)	95:5	47.4	Adam^35^	Mean squared error + custom	One-cycle LR	*r*
Camformers	CNN (ResNet^32^)	OHE [4:bases]	30	None	90:10	16.6	AdamW^36^	*L*_1_	Reduce LR on plateau	*r*, *ρ*
NAD	CNN + Transformer	GloVe^37^ [128]	0	None	90:10	15.5	AdamW^36^ + GSAM^52^	Smooth *L*_1_	Linear LR	*r*
wztr	CNN (ResNet^32^)	OHE [4:bases]	62	Input to model (concatenation with forward strand)	99:1	4.8	Adam^35^	Mean squared error	Reduce LR on plateau	*r*
High Schoolers Are All You Need (High Schoolers)	CNN + Transformer + multilayer perceptron	OHE [4:bases]	31	Model (RC parameter sharing)^51^	98:2	4.7	Adam^35^ + SWA^53^	Mean squared error	Multistep LR	*r*
BioNML	Vision Transformer^54^	OHE [4:bases]	30	Model (RC parameter sharing)^51^	86:14	78.7	Adamax^35^ + L2 regularizer	Huber	Multistep LR	*r,* CoD
BUGF	Transformer	OHE [6:bases/ N*/P*]	32	None	94:6	4.5	RAdam^55^	Multilabel focal loss^56^ + custom	None	*r*
mt	Gated recurrent unit^57^ + CNN	OHE [6:bases/ N*/P*]	62	Model (RC parameter sharing)^51^	99.8:0.2	3.1	Adam^35^	Binary cross-entropy	None	*r*, CoD^*#*^

*NC, if the sequence was present in more than one cell, 0 for all bases; otherwise, 1. RC, If the sequence is reverse-complemented, 1 for all bases; otherwise, 0. N, if a base is unknown, 1 for that base; otherwise 0. P, if a base has been padded to maintain fixed input length, 1 for that base; otherwise, 0. M, if a base is masked, 1 for that base; otherwise, 0. CoD, coefficient of determination.

^#^These teams used the metrics in a cross-validation setting to determine the optimal number of epochs for their models and ultimately saved the model weights after running for *n* epochs, without relying on validation metric scores. In contrast, other teams used validation metric scores to select the best-performing model.

The competing teams introduced several innovative approaches to solve the expression prediction problem. Autosome.org, the best-performing team, transformed the task into a soft-classification problem by training their network to predict a vector of expression bin probabilities, which was then averaged to yield an estimated expression level, effectively recreating how the data were generated in the experiment. They also used a distinct data-encoding method by adding channels to the traditional four-channel one-hot encoding (OHE) of the DNA sequence used by most teams. The two additional channels indicated (1) whether the sequence provided as input was likely measured in only one cell (which results in an integer expression value) and (2) whether the input sequence is being provided in the reverse complement orientation. Furthermore, Autosome.org’s model, with only 2 million parameters, was the model with the fewest parameters among the top ten submissions, demonstrating that efficient design can considerably reduce the necessary number of parameters. Autosome.org and BHI were distinct in training their final model on the entirety of the provided training data (that is, no sequences withheld for validation) for a prespecified number of epochs (determined previously using cross-validation using validation subsets). Unlock_DNA, the third best team, took a novel approach by randomly masking 5% of the input DNA sequence and having the model predict both the masked nucleotides and gene expression. This approach used the masked nucleotide predictions as a regularizer, adding a reconstruction loss to the model loss function, which stabilized the training of their large NN. BUGF, the ninth best team, used a somewhat similar strategy where they randomly mutated 15% of the sequence and calculated an additional binary cross-entropy loss predicting whether any base pair in the sequence had been mutated. The fifth best team, NAD, used GloVe^37^ to generate embedding vectors for each base position and used these vectors as inputs for their NN, whereas the other teams used traditional OHE DNA sequences. Two teams, SYSU-SAIL-2022 (11th) and Davuluri lab (16th), attempted to train DNA language models^38^ on the challenge data by pretraining a BERT (bidirectional encoder representations from transformers) language model^39^ on the challenge data and subsequently used the BERT embeddings to train an expression predictor.

### Test sequence subsets reveal model disparities

Analysis of model performance on the different test subsets revealed distinct and shared challenges for the different models. The top two models were ranked first and second (sometimes with ties) for each test subset regardless of score metric, showcasing that their superior performance could not be attributed to any single test subset (Fig. 1d,e). Furthermore, the rankings within each test subset sometimes differed between the Pearson score and Spearman score, reinforcing that these two measures capture performance in distinct ways (Fig. 1d,e).

**Extended Data Fig. 3:**
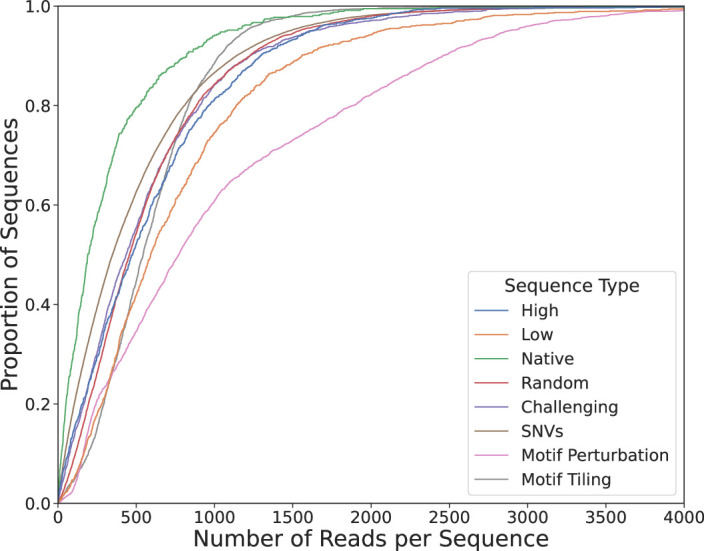
Library coverage differs between sequence subsets and is lowest for native sequences. Cumulative proportion (y-axis) of the number of reads per sequence (x-axis) for different sequence types (colours). Source data

While the ranking of models was similar for both random and native sequences, the differences in model performance were greater for native yeast sequences than random sequences. Specifically, performance differed between models by as much as 17.6% for native sequences but only 5% for random sequences (Pearson’s *r*^2^, Fig. 1f). Similarly, this difference was 9.6% (native) versus 2.7% (random) for Spearman’s *ρ* (Fig. 1g). This suggests that the top models learned more of the regulatory grammar that evolution has produced. Furthermore, the substantial discrepancy between performance on native and random sequences suggests that there is yet more regulatory logic to learn (although the native DNA has lower sequence coverage, presumably because of its higher repeat content, likely reducing data quality and predictability of this set; Extended Data Fig. 3).

Models were also highly variable in their ability to accurately predict variation within the extremes of gene expression. The cell sorter had a reduced signal-to-noise ratio at the lowest expression levels and the sorting bin placement could truncate the tails of the expression distribution^6,12^. Overall, model performance was most variable across teams in these subsets, suggesting that the challenge models were able to overcome these issues to varying degrees. For example, the median difference in Pearson’s *r*^2^ between the highest and lowest performance was ~48% for high-test and low-test subsets and 16% for the others (Fig. 1f,g).

**Extended Data Fig. 4:**
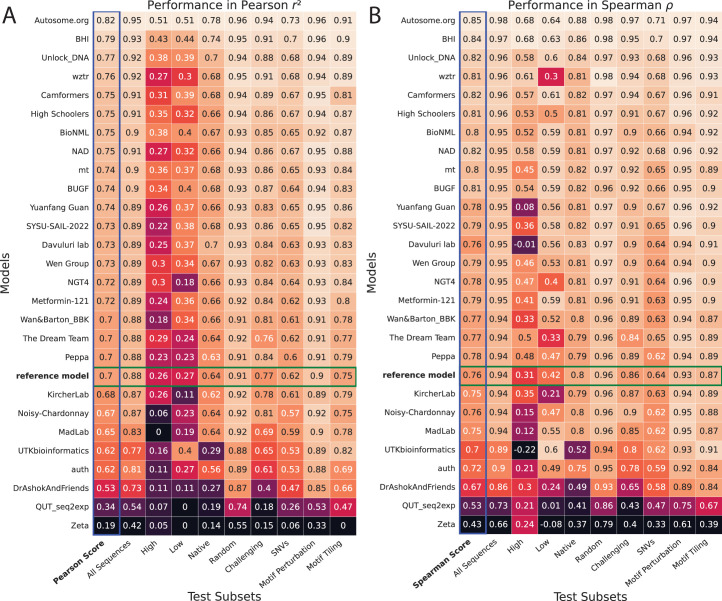
Performance of the teams in each test data subset. (**A,B**) Model performance (colour and numerical values) of each team (*y*-axes) in each test subset (*x*-axes), for (**A**) Pearson *r*^2^ and (**B**) Spearman ρ. Heatmap colour palettes are min-max normalized column-wise. Source data

**Extended Data Fig. 5:**
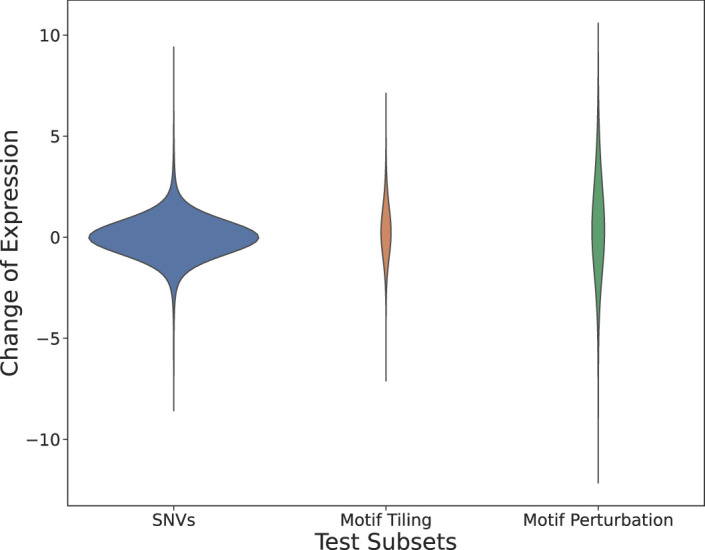
Expression changes in response to SNVs, motif tiling, and motif perturbation. Expression changes (*y*-axis) are biggest for motif perturbation, smallest for SNVs, and intermediate for motif tiling. Source data

The models also varied in their ability to predict expression differences between closely related sequences (Fig. 1de, ‘SNVs’, and Extended Data Figs. 4 and 5), with more substantial differences in model performance for subtler changes. Specifically, the percentage differences between best and worst in Pearson’s *r*^2^ and Spearman’s *ρ* were 6.5% and 4% for motif perturbation, 17.7% and 7% for motif tiling and 14.6% and 9.6% for SNVs, respectively, suggesting that the top-performing models better captured the subtleties of *cis*-regulation. This is consistent with our understanding of the subtlety of the impact; perturbing TFBSs (motif perturbations, where we mutate sequences strongly matching the cognate motif for an important TF or vary the number of binding sites) represented a comparatively large perturbation and could be predicted with simple models that capture the binding of these TFs and can count TFBS instances. However, when TFBSs are tiled across a background sequence, the same TFBS is present in every sequence and the model must have learned how its position affects its activity, in addition to capturing all the secondary TFBSs that are created or destroyed as the motif is tiled^13^. Lastly, SNVs are even harder to predict because nearly everything about the sequence is identical but for a single nucleotide that may affect the binding of multiple TFs in potentially subtle ways.

### Prix Fixe framework reveals optimal model configurations

**Fig. 2:**
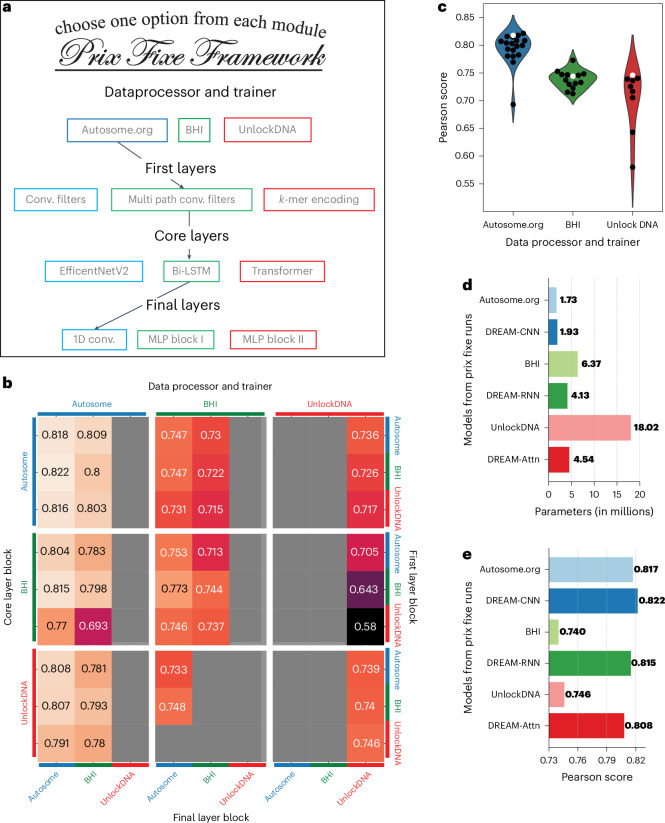
Dissecting the optimal model configurations through a Prix Fixe framework. **a**, The framework deconstructs each team’s solution into modules, enabling modules from different solutions to be combined. **b**, Performance in Pearson score from the Prix Fixe runs for all combinations of modules from the top three DREAM Challenge solutions. Each cell represents the performance obtained from a unique combination of core layer block (major rows, left), data processor and trainer (major columns, top), first layer block (minor rows, right) and final layer block (minor columns, bottom) modules. Gray cells denote combinations that were either incompatible or did not converge during training. **c**, Performance (Pearson score, *y* axis) of the three data processor and trainer modules (*x* axis and colors) for each Prix Fixe model including the respective module (individual points). Original model combinations are indicated by white points, while all other combinations are in black. **d**, Number of parameters (*x* axis) for the top three DREAM Challenge models (Autosome.org, BHI and UnlockDNA) along with their best-performing counterparts (based on core layer block), DREAM-CNN, DREAM-RNN and DREAM-Attn, in the Prix Fixe runs (*y* axis). **e**, As in **d**, but showing each model’s Pearson score (*x* axis). Source data

The top three solutions from the DREAM Challenge were distinguished both by their substantial improvement in performance compared to other models and their distinct approaches to data handling, preprocessing, loss calculations and diverse NN layers, encompassing convolutional, recurrent and self-attention mechanisms. To identify the factors underlying their performances, we developed a Prix Fixe framework that broke down each solution into distinct modules and, by selecting one of each module type, tested arbitrary combinations of the modules from each solution (Fig. 2a). We reimplemented the top three solutions within this framework and found that 45 of 81 possible combinations were compatible. We removed specific test time processing steps unique to each solution that were not comparable across solutions. Lastly, we retrained all compatible combinations using the same training and validation data, addressing the issue that some original solutions had used the entire dataset for training. Our approach facilitated a systematic and fair comparison of the individual contributions of different components to overall performance.

**Extended Data Fig. 6:**
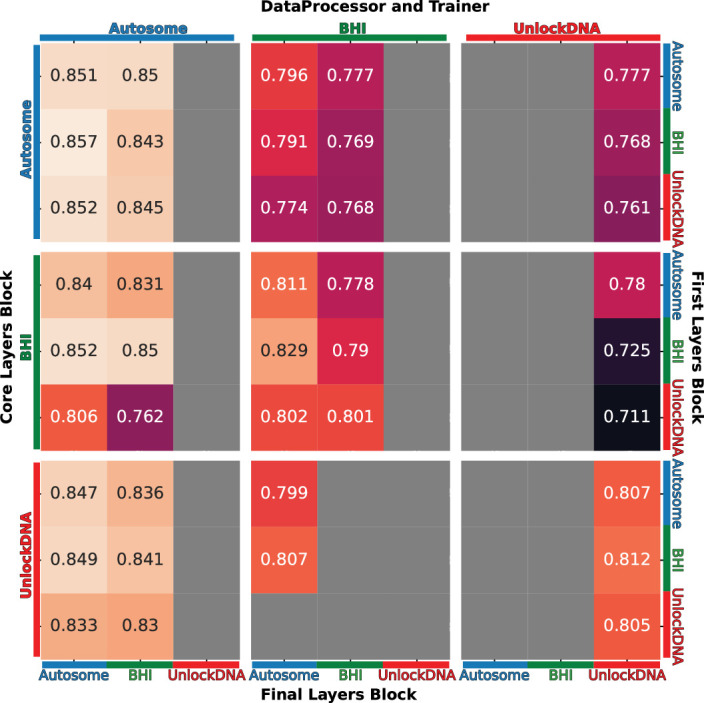
Performance in Spearman Score from the Prix Fixe runs for different possible combinations of the top three DREAM Challenge models. Modules are indicated on the axes, with Data Processor and Trainer models on the top *x*-axis, Final Layer Block on the bottom *x*-axis, Core Layers Block on the left *y*-axis, and First Layers Block on the right *y*-axis. Incompatible combinations and combinations that did not converge during training have been greyed out. Source data

**Extended Data Fig. 7:**
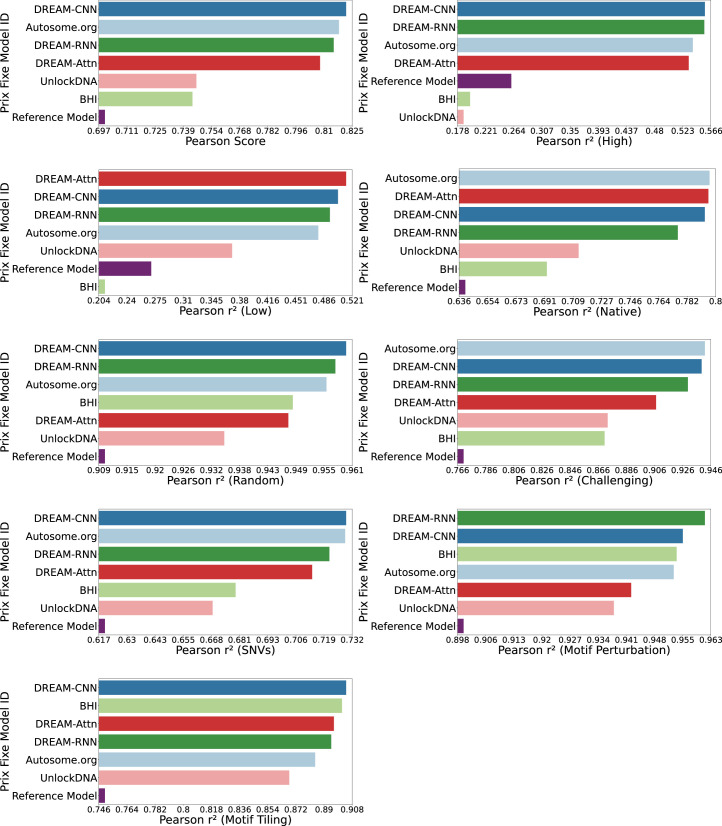
Performance comparison of top DREAM Challenge models and their best performing counterparts. Performance (*x*-axes) of the top three DREAM Challenge models (*y*-axes) Autosome.org, BHI, and UnlockDNA - along with their best-performing counterparts (based on Core Layers Block) for different test subsets. Source data

**Extended Data Fig. 8:**
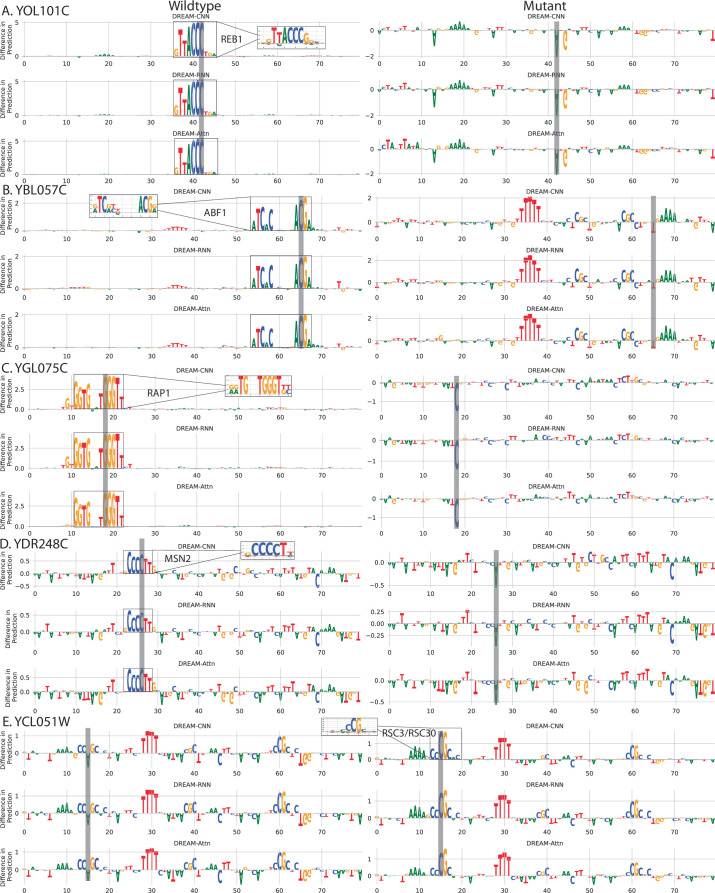
The DREAM-optimized models learn a very similar view of yeast cis-regulatory logic. **(A-E)** ISM scores (*y*-axes) for each nucleotide across each promoter region (*x*-axes and letters) for wild type (left) and SNV mutant (right) for yeast promoters (**A**) YOL101C, (**B**) YBL057C, (**C**) YGL075C, (**D**) YDR248C, and (**E**) YCL051W. Mutation locations are highlighted in grey. Probable transcription factor binding sites (**TFBSs**) altered by these mutations are marked with boxes, and the corresponding TF motifs are shown in the insets, identified using YeTFaSCo^40^. Source data

Our analysis revealed both the source of Autosome.org’s exceptional performance and the interplay of different model components, along with their potential for further optimization. The BHI and UnlockDNA NNs saw a notable improvement in performance when retrained using Autosome.org’s data processor and trainer (Fig. 2b,c and Extended Data Figs. 6 and 7). Moreover, each team’s model architecture could be optimized further, resulting in models that achieved better performance (Fig. 2c) using the same core blocks but with similar or fewer parameters (Fig. 2d). However, except for Autosome.org’s data processor and trainer module, no other module component dominated the others and their performance appeared to depend on what other modules they were combined with (Supplementary Fig. 1). For each core block of Autosome.org, BHI and UnlockDNA, we named the optimal Prix Fixe model as DREAM-CNN, DREAM-RNN and DREAM-Attn, respectively. The DREAM models learned a very similar view of the *cis*-regulatory logic as shown by the similar attribution scores (Extended Data Fig. 8) using in silico mutagenesis (ISM). Interestingly, in addition to agreeing on the large effects where recognizable consensus TFBSs were altered, the models also agreed on the smaller effects that varied in sign over 1–3 bp, which is too short to correspond to consensus TFBSs^40^, supporting the notion that the abundance of low-affinity binding sites has an important role in many *cis*-regulatory elements (CREs)^7,13,41^.

### Optimized models outperform the state of the art for other species and data types

**Fig. 3:**
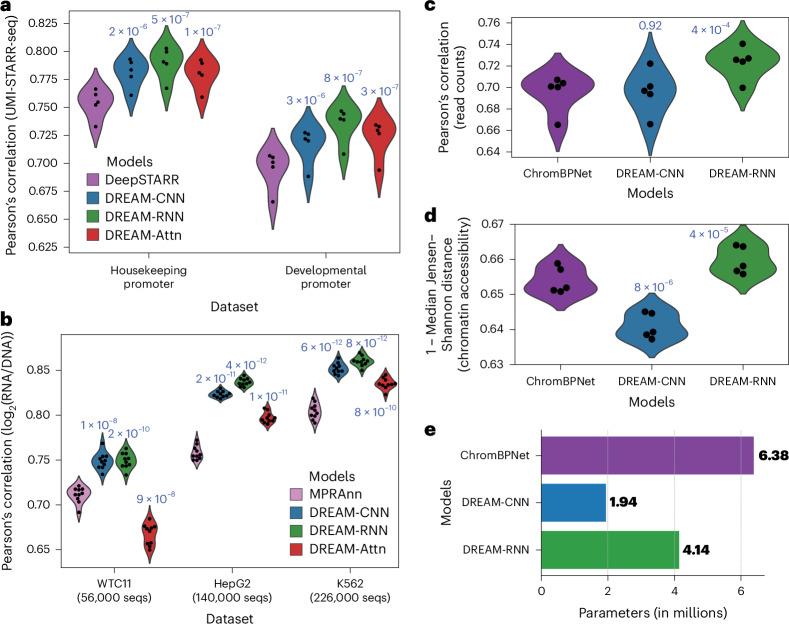
DREAM Challenge models beat existing benchmarks on *Drosophila* and human datasets. **a**, *D.* *melanogaster* STARR-seq^42^ prediction. Pearson’s correlation for predicted versus actual enhancer activity for held-out data (*y* axis) for two different transcriptional programs (*x* axis) for each model (colors). **b**, Human MPRA^45^ prediction. Pearson correlation for predicted versus actual expression for held-out data (*y* axis) for MPRA datasets from three distinct human cell types (*x* axis) for each model (colors). **c**,**d**, Human accessibility (bulk K562 ATAC-seq)^46,49^ prediction. For each model (*x* axis and colors), model performance (*y* axes) is shown in terms of both Pearson’s correlation for predicted versus actual read counts per element (**c**) and 1 − median Jensen–Shannon distance for predicted versus actual chromatin accessibility profiles across each element (**d**). In **a**–**d**, points represent folds of cross-validation, performance is evaluated on held-out test data and *P* values determined by *t*-tests (paired, two-sided) comparing the previous state-of-the-art model to the optimized models are shown above the model performance distributions. **e**, Comparison of the number of parameters (*x* axis) for different models used in chromatin accessibility prediction task. Source data

To determine whether the model architectures and training strategies we optimized on yeast data would generalize to other species, we next applied them to *Drosophila melanogaster* and human datasets on a diverse set of tasks. First, we tested their ability to predict gene regulatory activity measured in *D.* *melanogaster* (in the context of a developmental and a housekeeping promoter) in a self-transcribing active regulatory region sequencing (STARR-seq) massively parallel reporter assay (MPRA). This fundamentally represents the same sequence-to-expression problem the models were designed to solve, despite the different organism (*Drosophila* versus yeast), experimental measurement approach (RNA sequencing versus cell sorting), longer sequence (249 bp versus 150 bp), smaller datasets (~500,000 versus 6.7 million) and the transition from a single-task to a multitask framework (two promoter types). We compared the DREAM-optimized models to DeepSTARR^42^, a state-of-the-art CNN model based on the Basset^20^ architecture and specially developed for predicting the data we used in this benchmark (STARR-seq with unique molecular identifier integration (UMI-STARR-seq)^43^ in *D.* *melanogaster* S2 cells^42,44^). For a robust comparison, we trained the models using cross-validation and always evaluated on the same held-out test data (Methods). Our models consistently outperformed DeepSTARR across both developmental and housekeeping transcriptional programs (Fig. 3a), with the DREAM-RNN’s model performance surpassing that of DREAM-CNN and DREAM-Attn.

To further validate the generalizability of our models, we next trained the DREAM-optimized models on lentivirus-based MPRAs (lentiMPRAs) that tested CREs across three human cell types: hepatocytes (HepG2), lymphoblasts (K562) and induced pluripotent stem cells (WTC11)^45^. Here, our models had to capture more complex regulatory activity from vastly smaller datasets (~56,000–226,000 versus 6.7 million). We compared the models against MPRAnn^45^, a CNN model optimized for these specific datasets (Methods). All models were trained using cross-validation and evaluated on held-out test data in the same way that MPRAnn was originally trained^45^. The DREAM-optimized models substantially outperformed MPRAnn, with the performance difference widening with more training data (Fig. 3b). The only exception was DREAM-Attn, which did not outperform MPRAnn on the smallest dataset (WTC11; 56,000 sequences). Again, DREAM-RNN demonstrated the best performance among our models, especially for larger datasets.

To evaluate the models on a distinct prediction task that still relates to CRE function, we evaluated our optimized models on the task of predicting open chromatin. Specifically, we compared our optimized models to ChromBPNet^46–48^, a BPNet-based^16^ model that predicts assay for transposase-accessible chromatin with sequencing (ATAC-seq) signals across open chromatin regions. Here, the input DNA sequences were ~14 times longer than the yeast promoters on which the DREAM models were optimized (2,114 versus 150 bp) and the models were now tasked with simultaneously predicting the overall accessibility (read counts) and accessibility profile (read distribution) for a central 1,000-bp section, rather than predicting a single expression value. While DREAM-Attn could not be trained because the memory requirement for the attention block became too large with such a long input sequence, we trained and evaluated the other DREAM-optimized models and ChromBPNet on K562 bulk ATAC-seq data^49^ (Methods). DREAM-RNN outperformed ChromBPNet substantially in predictions of both read count and chromatin accessibility (Fig. 3c,d), highlighting the adaptability of our models even on substantially different *cis*-regulatory data types. DREAM-CNN, on the other hand, performed on par with ChromBPNet^46^ in predictions of read count (Fig. 3c) but was less effective in predicting chromatin accessibility profiles (Fig. 3d).

**Extended Data Fig. 9:**
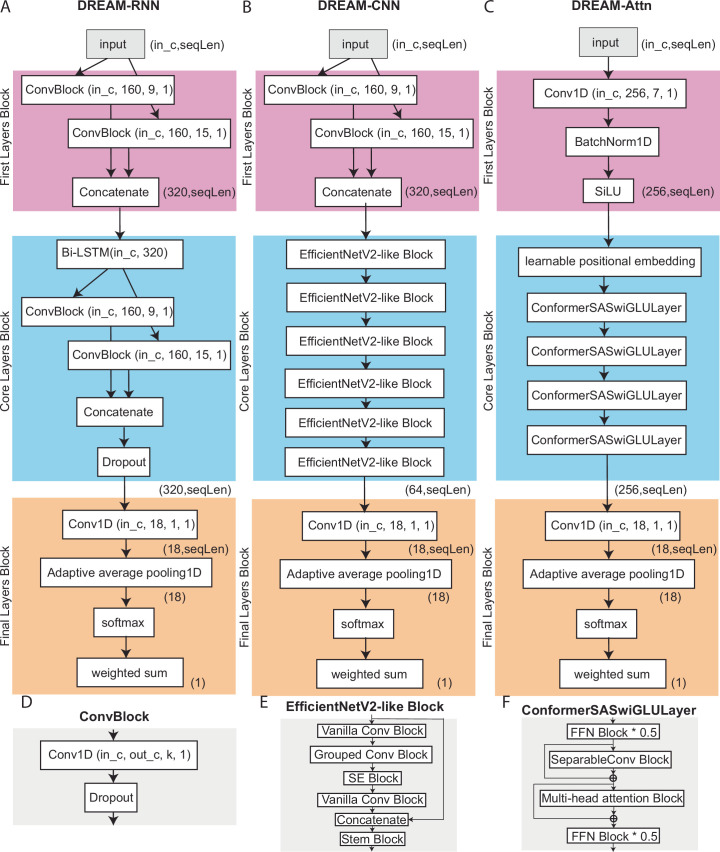
NN architecture diagrams of the DREAM-optimized models. **(A-C)** High level illustration of the (**A**) DREAM-RNN, (**B**) DREAM-CNN, and (**C**) DREAM-Attn models. (**D-F**) High level illustration of different network blocks used within the core layers of (**A-C**). The Vanilla Conv Block, Grouped Conv Block, SE Block, Stem Block, FFN Block, SeparableConv Block, and Multi-head attention Block are described in detail in^66^ and^69^.

Notably, the architectures and training paradigms of the DREAM-optimized models were changed minimally for these evaluations (Extended Data Fig. 9). The components that could not accommodate the data were discarded (for example, the input-encoding channel denoting singleton observations was not compatible to STARR-seq, MPRA and ATAC-seq data; Methods). The only other modifications made were required for the prediction head to predict the new task (for example, the final layer block architecture and using task-specific loss functions; Methods) or to adapt to the smaller number of training sequences compared to the DREAM dataset (reducing the batch size and/or maximum learning rate (LR); Methods). Importantly, DREAM-RNN outperformed the other Prix Fixe optimized models in all of these secondary benchmarks (Fig. 3a–d), highlighting its excellent generalizability.

## Discussion

The Random Promoter DREAM Challenge 2022 presented a unique opportunity for participants to propose novel model architectures and training strategies for modeling regulatory sequences. The participants trained sequence-to-expression models on millions of random regulatory DNA sequences and their corresponding expression measurements. A separate set of designed sequences were used to evaluate these models and test their limits. Remarkably, 19 models from the DREAM Challenge outperformed the previous state of the art^22^ (Extended Data Fig. 4), with the majority using unique architectures and training strategies. To systematically analyze how model design choices impact their performance, we developed the Prix Fixe framework, where models were abstracted to modular parts, enabling us to combine modules from different submissions to identify the key contributors to model performance. We applied the Prix Fixe framework to the top three models from the challenge that varied substantially in their NN architectures (CNN, RNN and self-attention) and training strategies and were able to construct improved models in each case.

The training strategies for NNs had as notable an impact on model performance as the network architectures themselves (Fig. 2c and Extended Data Fig. 7). In the Prix Fixe runs, training the network to predict expression as distributions using soft classification rather than as precise values helped models capture more of *cis*-regulation. These findings argue for a balanced focus not only on network architectures but also on the optimization of training procedures and redefinition of prediction tasks.

**Extended Data Fig. 10:**
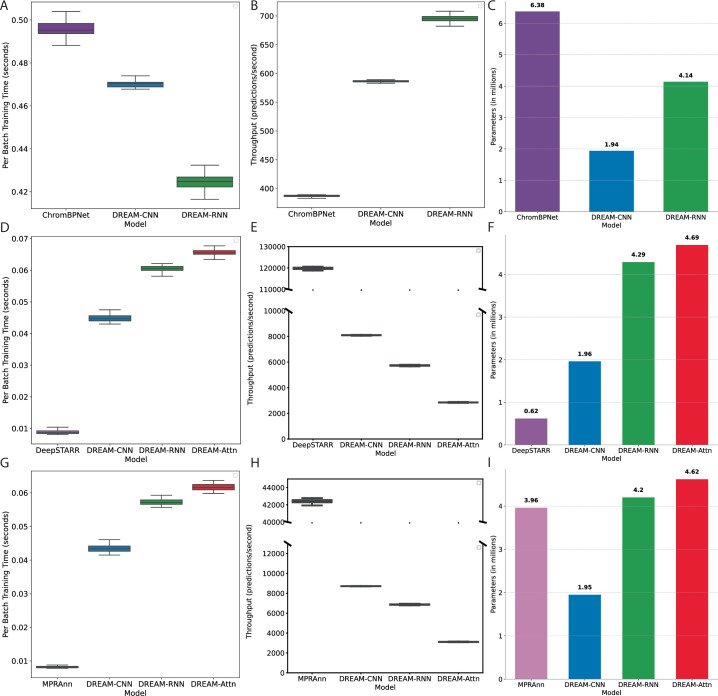
Comparative analysis of computational efficiency (per batch training time and throughput) and capacity (number of parameters) across different models. **(A, D, G)** Per batch training time in seconds (*y*-axes), **(B,E,H)** throughput in predictions per second (*y*-axes), and **(C, F, I)** number of parameters (*y*-axes) for the models (*x*-axes, colours) applied to (**A-C**) human ATAC-seq, (**D-F**) Drosophila STARR-seq, and (**G-I**) human MPRA dataset. Boxplots represent the distribution of measurements for training time per batch (A, D, G) and throughput (B, E, H), which were repeated 50 times to ensure reliability. Source data

Notably, the top-performing models from the DREAM Challenge demonstrated that simpler NN architectures with fewer parameters, if optimized well, can effectively capture much of the activity of individual CREs. Three of the top five submissions did not use transformers, including the best-performing team (which also had the fewest parameters of the top ten). Using our Prix Fixe framework, we successfully designed models that not only consisted of similar or fewer parameters but also achieved superior performance compared to their counterparts (Fig. 2d,e). Furthermore, these DREAM-optimized models consistently outperformed previous state-of-the-art models on other *cis*-regulatory tasks, despite having comparable (and often fewer) parameters than previous models (Figs. 1d and 3e and Extended Data Fig. 10). While genomics model design requires consideration of the nature of the task (for example, enhancer-gene regulation necessarily requires the ability to capture long-range interactions), our findings highlight that building better models mostly depends on effective optimization rather than simply increasing model capacity. However, building better models may come with increased computational burden as the biochemistry is approximated with finer resolution (Extended Data Fig. 10).

In the DREAM Challenge, we observed varied results across test subsets that illustrate the complexity in evaluating *cis*-regulatory models effectively. For instance, performance on random sequences, which were in the same domain as the training data (also random sequences), was relatively uniform (Fig. 1d,e). Conversely, shifting the domain to native sequences highlighted the disparities between models, as the relative frequencies of various regulatory mechanisms likely differ, a consequence of their evolutionary origin (Fig. 1d,e). This indicates that a model that excels in modeling overall *cis*-regulation may still perform poorly for sequences involving certain regulatory mechanisms (for example, cooperativity in evolved sequences) that are difficult to learn from the training data, leading to incorrect predictions of biochemical mechanisms and variant effects for sequences that use these mechanisms. This emphasizes the importance of multifaceted evaluation of genomics models^50^ and designing specific datasets that test the limits of these models.

To continually improve genomics models, there is a need for standardized, robust benchmarking datasets. The DREAM Challenge dataset addresses this need and the impact that such standardized datasets can have was demonstrated by the generalizability of DREAM-optimized models across different *Drosophila* and human datasets and tasks without additional model tuning. Nonetheless, it should be noted that the models stemming from this challenge explored only a fraction of the possible design space and are likely to be improved upon. Furthermore, performance of the DREAM-optimized models can be optimized for different datasets by tailoring hyperparameters of these models to the dataset in question or by using ensembles of the models. Our dataset accompanied by the Prix Fixe framework stands as a valuable resource for the continued exploration and development of innovative NN architectures and training methodologies specifically crafted for DNA sequences. Furthermore, the modular nature and proven generalizability of the DREAM-optimized models will enable other researchers to easily apply them to other genomics problems.

## Methods

### Designing the test sequences

High-expression and low-expression sequences were designed using DEAP^58^ with the mutation probability and the two-point crossover probability set to 0.1, selection tournament size of 3, initial population size of 100,000 and the genetic algorithm run for ten generations, using the predictions of a CNN trained on random yeast promoter sequences as the fitness function^22^. Native test subset sequences were designed by sectioning native yeast promoters into 80-bp fragments^13^. Random sequences were sampled from a previous experiment where the tested DNA was synthesized randomly (as in the training data) and quantified^13^. Challenging sequences were designed by maximizing the difference between the expressions predicted by a CNN model^22^ and a biochemical model (a type of physics-informed NN)^13^; these sequences represented the pareto front of the differences in expression between models when optimizing populations of 100 sequences at a time for 100 generations using a genetic algorithm with a per-base mutation rate of 0.02 and recombination rate of 0.5 using DEAP^58^ and a custom script (GASeqDesign.py^59^). Most of the SNVs represented sequence trajectories from Vaishnav et al.^22^ but also included random mutations added to random, designed and native promoter sequences. Motif perturbation included Reb1 and Hsf1 perturbations. Sequences with perturbed Reb1 binding sites were created by inserting Reb1 consensus binding sites (strong or medium affinity; sense and reverse complement orientations) and then adding 1–3 SNVs to each possible location of each motif occurrence and inserting canonical and mutated motif occurrence into ten randomly generated sequences at position 20 or 80. Sequences with Hsf1 motif occurrence were designed by tiling random background sequences with 1–10 Hsf1 monomeric consensus sites (ATGGAACA), added sequentially from both right and left of the random starting sequences, added individually within each of the possible eight positions or similarly tiling or inserting 1–5 trimeric Hsf1 consensus sites (TTCTAGAANNTTCT). The motif tiling test subset sequences were designed by embedding a single consensus for each motif (poly(A), AAAAA; Skn7, GTCTGGCCC; Mga1, TTCT; Ume6, AGCCGCC; Mot3, GCAGGCACG; Azf1, TAAAAGAAA) at every possible position (with the motif contained completely within the 80-bp variable region) and orientation for three randomly generated background sequences^13^.

### Quantifying promoter expression

High-complexity random DNA libraries that comprised the training data were created using Gibson assembly to assemble a double-stranded random DNA insert into a dual reporter vector yeast_DualReporter (AddGene, 127546). The random DNA insert was created by annealing a complementary primer sequence and extending to double strand using Phusion polymerase master mix (New England Biolabs) and gel-purifying before cloning. The random DNA was inserted between distal (GCTAGCAGGAATGATGCAAAAGGTTCCCGATTCGAACTGCATTTTTTTCACATC) and proximal (GGTTACGGCTGTTTCTTAATTAAAAAAAGATAGAAAACATTAGGAGTGTAACACAAGACTTTCGGATCCTGAGCAGGCAAGATAAACGA) promoter regions. The random promoter library in *Escherichia coli* theoretically contained about 74 million random promoters (estimated by dilution and plating) and was transformed into S288c (*ΔURA3*) yeast yielding 200 million transformants, which were selected in SD-Ura medium. Then, 1 L of Chardonnay grape must (filtered) was inoculated with the pool to an initiate an optical density at 600 nm (OD_600_) of 0.05 and grown at room temperature without continual shaking, with the culture diluted as needed with fresh Chardonnay grape must to maintain the OD below 0.4, for a total growth time of 48 h and having undergone >5 generations. Before each OD measurement, the culture was gently agitated to decarbonate it, waiting for the resulting foam to die down before agitating again and continuing until no more bubbles were released. Before sorting, yeasts were spun down, washed once in ice-cold PBS, resuspended in ice-cold PBS, kept on ice and then sorted by log_2_(red fluorescent protein (RFP)/YFP) signal (using mCherry and green fluorescent protein absorption and emission) on a Beckman-Coulter MoFlo Astrios, using the constitutive RFP under pTEF2 regulation to control for extrinsic noise^13^. Cells were sorted into 18 uniform bins, in three batches of six bins each. After sorting, cells from each bin were spun down and resuspended in SC-Ura and then grown for 2–3 days, shaking at 30 °C. Plasmids were isolated, the promoter region was amplified, Nextera adaptors and multiplexing indices were added by PCR and the resulting libraries were sequenced, with sequencing libraries pooled and sequenced on an Illumina NextSeq using 2 × 76-bp paired-end reads with 150-cycle kits. The designed (test) experiment was performed similarly but the library was amplified by PCR from a Twist oligo pool and the *E.* *coli* transformation complexity was only 105, over 10× coverage of the library.

To obtain sequence–expression pairs for random promoter sequences, the paired-end reads representing both sides of the promoter sequence were aligned using the overlapping sequence in the middle, constrained to have 40 ± 15 bp of overlap, discarding any reads that failed to align well within these constraints^13^. To collapse related promoters into a single representative sequence, we aligned the sequences observed in each library to themselves using Bowtie2 (ref. ^60^), creating a Bowtie database containing all unique sequences observed in the experiment (default parameters) and aligning these same sequences, which allowed for multimapping reads (parameters included ‘-N 1 -L 18 -a -f -no-sq -no-head -5 17 -3 13’. Any sequences that aligned to each other were assigned to the same cluster, which were merged using the sequence with the most reads as the ‘true’ promoter sequence for each cluster. Expression levels for each promoter sequence were estimated as the weighted average of bins in which the promoter was observed^13^. For the designed (test) library, we instead directly aligned reads to a Bowtie database of the sequences we ordered to quantify and estimated their expression levels using MAUDE^61^, with the read abundance in each sorting bin as input, and estimating the initial abundance of each sequence as the average relative abundance of that sequence across all bins.

### Competition rules

1. Only the provided training data could be used to train models. Models had to train from scratch without any pretraining on external datasets to avoid overfitting to sequences present in the test data (for example, some sequences in the test data were derived from extant yeast promoters).
2. Reproducibility was a prerequisite for all submissions. The participants had to provide the code and instructions to reproduce their models. We retrained the top-performing solutions to validate their performance.
3. Augmenting the provided training data was allowed. Pseudolabeling the provided test data was not allowed. Using the test data for any purpose during training was not allowed.
4. Ensembles were not allowed.

Detailed information on the competition and its guidelines can be found on the DREAM Challenge webpage (https://www.synapse.org/#!Synapse:syn28469146/wiki/617075).

### Performance evaluation metric

We calculated Pearson’s *r*^*2*^ and Spearman’s *ρ* between predictions and measurements for each sequence subset. The weighted sum of each performance metric across promoter types yielded our two final performance measurements, which we called the Pearson score and Spearman score.

$${\rm{Pearson}}\; {\rm{score}}=\mathop{\sum }\limits_{i\,=\,0}^{\rm{subsets}}{w}_{i}\times {\rm{Pearson}}\;{{r}^{2}}_{i}\,/\,\mathop{\sum }\limits_{i\,=\,0}^{\rm{subsets}}{w}_{i}$$

$${\rm{Spearman}}\; {\rm{score}}=\mathop{\sum }\limits_{{i}=\,0}^{\rm{subsets}}{w}_{{i}} \times {\rm{Spearma}{n}}_{i}/\mathop{\sum }\limits_{{i}=\,0}^{\rm{subsets}}{w}_{i}$$

Here, *w*_*i*_ is the weight used for the *i*th test subset (Table 1). ${\rm{Pearson}}\;{{r}^{2}}_{i}$ and ${\rm{Spearma}{n}}_{i}$ are, respectively, the square of the Pearson coefficient and the Spearman coefficient for sequences in the *i*th subset.

### Bootstrapping analysis of model performance

To determine the relative performance of the models, we performed a bootstrapping analysis. Here, we sampled 10% of the test data 10,000 times and, for each sample, calculated the performance of each model and the rankings of the models for both Pearson and Spearman scores. We averaged the ranks from both metrics to decide their final ranks.

### Description of the approaches used by the participants

An overview of the approaches used by the participants in the challenge is provided in the Supplementary Information.

### Prix Fixe framework

The Prix Fixe framework, implemented in Python and Pytorch, facilitated the design and training of NNs by modularizing the entire process, from data-preprocessing to prediction, enforcing specific formats for module inputs and outputs to allow integration of components from different approaches. The different modules in the Prix fixe framework are described below.

#### Data processor and trainer

The data processor class is dedicated to transforming raw DNA sequence data into a usable format for subsequent NN training. The data processor can produce an iterable object, delivering a dictionary containing, a feature matrix ‘*x*’ (input to the NN) and a target vector ‘*y*’ (expected output). Additional keys can be included to support extended functionalities. Moreover, the data processor can provide essential parameters to initiate NN blocks, such as determining the number of channels in the first layer.

The trainer class manages the training of the NN. It processes batches of data from the data processor and feeds them into the NN. It computes auxiliary losses, if necessary, alongside the main losses from the NN, facilitating complex loss calculation during training.

#### Prix Fixe net

This module embodies the entirety of the NN architecture:

(i) First layer block: This constitutes the primordial layers of the network. They may include initial convolutional layers or facilitate specific encoding mechanisms such as *k*-mer encoding for the input.
(ii) Core layer block: This represents the central architecture components, housing elements such as residual connections, LSTM mechanisms and self-attention. The modular construction of this block also allows for versatile combinations, such as stacking a residual CNN block with a self-attention block.
(iii) Final layer block: This phase narrows the latent space to produce the final prediction, using layers such as pooling, flattening and dense layers. It computes the prediction and outputs it alongside the loss.

For all three blocks, the standard input format is (batch, channels, seqLen). The first two blocks yield an output in a consistent format (batch, channels, seqLen), whereas the last block delivers the predicted expression values. Each block can propagate its own loss. The whole framework is implemented in PyTorch.

To ensure fair comparison across solutions in the Prix Fixe framework, we removed specific test time processing steps that were unique to each solution. We divided the DREAM Challenge dataset into two segments, allocating 90% sequences for training and 10% for validation. Using these data, we retrained all combinations that were compatible within the framework. Of the 81 potential combinations, we identified 45 as compatible and 41 of these successfully converged during training. Because of graphics processing unit (GPU) memory constraints, we adjusted the batch sizes for certain combinations.

### DREAM-optimized models from Prix Fixe runs

#### Data processor and trainer

Promoter sequences were extended at the 5′ end using constant segments from the plasmids to standardize to a length of 150 bp. These sequences underwent OHE into four-dimensional vectors. ‘Singleton’ promoters, observed only once across all bins, were categorized with integer expression estimates. Considering the potential variability in these singleton expression estimates, a binary ‘is_singleton’ channel was incorporated, marked as 1 for singletons and 0 otherwise. To account for the diverse behavior of regulatory elements on the basis of their strand orientation relative to transcription start sites, each sequence in the training set was provided in both its original and reverse complementary forms, identified using the ‘is_reverse’ channel (0 for original and 1 for reverse complementary). Consequently, the input dimensions were set at (batch, 6, 150).

The model’s training used the AdamW optimizer, set with a weight_decay of 0.01. The maximum LR of 0.005 was chosen for most blocks, while a conservative rate of 0.001 was applied to attention blocks because of the inherent sensitivity of self-attention mechanisms to higher rates. This LR was scheduled by the one-cycle LR policy^62^, which featured two phases and used the cosine annealing strategy. Training data were segmented into batches of size 1,024, with the entire training procedure spanning 80 epochs. Model performance and selection were based on the highest Pearson’s *r* value observed in the validation dataset.

During prediction, the data processing mirrored the data processor apart from setting ‘is_singleton’ to 0. Predictions for both the original and reverse complementary sequences were then averaged.

#### Prix Fixe net

##### DREAM-CNN

First layer block: The OHE input was processed through a one-dimensional (1D) CNN. Drawing inspiration from DeepFam^63^, convolutional layers with kernel sizes of 9 and 15 were used, mirroring common motif lengths as identified by ProSampler^64^. Each layer had a channel size of 256, used rectified linear unit activation and incorporated a dropout rate of 0.2. The outputs of the two layers were concatenated along the channel dimension.

Core layer block: This segment contained six convolution blocks whose structure was influenced by the EfficientNet architecture. The segment contained modifications such replacing depth-wise convolution with grouped convolution, using squeeze and excitation (SE) blocks^65^ and adopting channel-wise concatenation for residual connections. The channel configuration started with 256 channels for the initial block, followed by 128, 128, 64, 64, 64 and 64 channels^66^.

Final layer block: The final block consisted of a single point-wise convolutional layer followed by channel-wise global average pooling and SoftMax activation.

##### DREAM-RNN

First layer block: Same as DREAM-CNN.

Core layer block: The core used a Bi-LSTM, designed to capture motif dependencies. The LSTM’s hidden states had dimensions of 320 each, resulting in 640 dimensions after concatenation. A subsequent CNN block, similar to the first layer block, was incorporated.

Final layer block: Same as DREAM-CNN.

##### DREAM-Attn

First layer block: This segment was a standard convolution with kernel size 7, followed by BatchNorm^67^ and sigmoid linear unit activation^68^.

Core layer block: This block used the Proformer^69^, a Macaron-like transformer encoder architecture, which uses two half-step feed-forward network (FFN) layers at the start and end of the encoder block. Additionally, a separable 1D convolution layer was integrated after the initial FFN layer and before the multihead attention layer.

Final layer block: Same as DREAM-CNN and DREAM-RNN.

### Human MPRA models

Within each of the three large-scale MPRA libraries, every sequence and its corresponding reverse complement were grouped together and these pairs were then distributed into ten distinct cross-validation folds to ensure that both the forward and the reverse sequences resided within the same fold. DREAM-CNN, DREAM-RNN, DREAM-Attn and MPRAnn were trained using nine of these ten folds, reserving one fold to evaluate the model’s performance. For every held-out test fold, nine models were trained, with one fold being dedicated for validation purposes while the remaining eight acted as training folds. Subsequent predictions from these nine models were aggregated, with the average being used as the final prediction for the held-out test data.

The MPRAnn architecture^45^ was trained with an LR of 0.001, an early stopping criterion with patience of 10 on 100 epochs, a batch size of 32 and the Adam optimizer with a mean squared error loss function. For DREAM-CNN, DREAM-RNN and DREAM-Attn, components that could not accommodate Agarwal et al.’s data were discarded. For instance, the ‘is_singleton’ channel is not relevant for MPRA data and loss calculation was performed using the mean squared error (as in MPRAnn) in place of Kullback–Leibler divergence because of the infeasibility of transitioning the problem to soft classification. MPRAnn used a much smaller batch size than our DREAM-optimized trainer (32 versus 1,024); thus, we reduced it to be the same as MPRAnn. No other alterations were made to either the model’s structure or the training paradigm.

### Drosophila UMI-STARR-seq models

The DeepSTARR architecture^42^ was trained with an LR of 0.002, an early stopping criterion with patience of 10 on 100 epochs, a batch size of 128 and the Adam optimizer with a mean squared error loss function. For DREAM-CNN, DREAM-RNN and DREAM-Attn, we used the exact setting as used for human MPRA datasets but with the output layer modified to predict two values corresponding to expression with housekeeping and developmental promoters and the loss calculated as the sum of the mean squared errors for each output (as in DeepSTARR).

Only the five largest *Drosophila* chromosomes (Chr2L, Chr2R, Chr3L, Chr3R and ChrX) were used as test data. For every held-out test chromosome, the remaining sequences were distributed into ten distinct train–validation folds and DREAM-CNN, DREAM-RNN, DREAM-Attn and DeepSTARR models (ten of each) were trained. Subsequent predictions from these ten models were aggregated, with the average being used as the final prediction for the held-out test chromosome.

### Human chromatin accessibility models

We used five separate train–validation–test splits as proposed in a previous study^46^ for ATAC-seq experiments on the human cell line K56249. For each of these partitions, we first trained five different bias models, one per fold, which were designed to capture enzyme-driven biases present in ATAC-seq profiles. Subsequently, ChromBPNet, DREAM-CNN and DREAM-RNN models were trained for each fold, using the same bias models. For DREAM-CNN and DREAM-RNN, the prediction head from ChromBPNet (1D convolution layer, cropping layer, average pooling layer and a dense layer) was used in the final layer block to predict the accessibility profile and read counts. Input-encoding channels for is_singleton and is_rev were omitted. We modified the DREAM-optimized trainer in this case to use the same batch size as ChromBPNet (from 1,024 to 64) and a reduced maximum LR (from 5 × 10^−3^ to 5 × 10^−^^4^). No other alterations were made to either the model’s structure or the training paradigm.

For this task, we reimplemented DREAM-CNN and DREAM-RNN architectures in TensorFlow to ensure that all models had same bias models. This methodological choice came at the cost of having to leave some components (input encoding, AdamW optimizer, etc.) out of the DREAM-optimized data processor and trainer. However, it ensured uniformity across models, leading to an unbiased comparison across the different architectures.

### ISM

The ISM scores for DNA sequences were obtained by creating all possible single-nucleotide mutations of each sequence and calculating the change in predicted expression relative to the original sequence. A single ISM score for each position was then determined by averaging the mutagenesis scores across nucleotides at that position.

### Average training time per batch and throughput

We measured the training time per batch for the human ATAC-seq dataset with a batch size of 64 and on two other datasets with a batch size of 32. Throughput was determined by measuring how many data points each model could predict per second (without backpropagation). Starting with a batch size of 32, we doubled the batch size incrementally (64, 128, 256, etc.) and recorded the throughput at each stage until the maximum batch size supportable by the GPU was reached, which was then used to calculate throughput. We processed 5,000 batches for each model to calculate the average training time per batch and processed 100 batches for throughput. The calculations for both training time per batch and throughput were repeated 50 times to ensure reliability and the distribution of these measurements is presented as a box plot in Extended Data Fig. 10. All tests were conducted using an NVIDIA V100 16-GB GPU, ensuring consistency in the computational resources across all experiments.

### Reporting summary

Further information on research design is available in the Nature Portfolio Reporting Summary linked to this article.

## Online content

Any methods, additional references, Nature Portfolio reporting summaries, source data, extended data, supplementary information, acknowledgements, peer review information; details of author contributions and competing interests; and statements of data and code availability are available at 10.1038/s41587-024-02414-w.

## Supplementary information

Supplementary Information Supplementary Information, Table 1 and Fig. 1.

Reporting Summary

## Source data

Source Data Fig. 1 Statistical source data.

Source Data Fig. 2 Statistical source data.

Source Data Fig. 3 Statistical source data.

Source Data Extended Data Fig. 1 Statistical source data.

Source Data Extended Data Fig. 2 Statistical source data.

Source Data Extended Data Fig. 3 Statistical source data.

Source Data Extended Data Fig. 4 Statistical source data.

Source Data Extended Data Fig. 5 Statistical source data.

Source Data Extended Data Fig. 6 Statistical source data.

Source Data Extended Data Fig. 7 Statistical source data.

Source Data Extended Data Fig. 8 Statistical source data.

Source Data Extended Data Fig. 10 Statistical source data.

## Data Availability

Data generated for this study are available from the National Center of Biotechnology Information Gene Expression Omnibus (GEO) under accession number GSE254493. The processed datasets are available from Zenodo (10.5281/zenodo.10633252)^70^. The Drosophila STARR-seq data are available from the GEO under accession number GSE183939. The human MPRA dataset is available from Zenodo (10.5281/zenodo.8219231)^71^. The human ATAC-seq data is available from the GEO under accession number GSE170378. Source data are provided with this paper.

## Code availability

Open-source code for our models is available from GitHub (https://github.com/de-Boer-Lab/random-promoter-dream-challenge-2022).
