# Supplementary material for: A community effort to optimize sequence-based deep learning models of gene regulation
Source: Nat Biotechnol. 2024 Oct 11;43(8):1373–83. doi: 10.1038/s41587-024-02414-w (PMC12339383; doi:10.1038/s41587-024-02414-w)
Supplement: Supplementary file 1 — Supplementary Information, Table 1 and Fig. 1. [file 41587_2024_2414_MOESM1_ESM.pdf]

nature biotechnology

Supplementary information https://doi.org/10.1038/s41587-024-02414-w

A community effort to optimize sequence-based deep learning models of gene regulation

In the format provided by the authors and unedited

Supplementary Methods

Description of the approaches used by the participants

In this section, we present an overview of the approaches employed by the participants in the challenge. For the top-performing teams, we provide a detailed description of their methodologies, while for the remaining teams, we offer a concise overview without repeating any details that have already been discussed for other teams.

Autosome.org: The team reformulated the initial regression task as a soft-classification problem by replacing the initial target expression value with a vector of 18 probabilities corresponding to individual bins, assuming that they can be deduced from the normal distribution with mean and variance equal to (expression + 0.5) and 0.5, respectively. To obtain a predicted expression value for a sequence during the validation step, the predicted probabilities were multiplied by their bin numbers. They used one-hot encoding for the promoter sequences, where they added a separate binary channel explicitly marking the objects with integer (and thus likely imprecise) expression measurements. They also augmented the dataset with the reverse complementary sequences and added a separate binary channel to denote the supplied strand explicitly (forward or reverse complementary). The team also highlighted the impact of the training regime on the final performance, specifically, the advantage of using the OneCycleLR scheduler coupled with the AdamW optimizer. The proposed model was based on a fully-convolutional network inspired by EfficientNetV2 (1). The following architectural choices were used in the final model: (i) grouped convolution (2) instead of the depthwise convolution of the original EfficientNetV2, (ii) the standard residual blocks were substituted with residual channel-wise concatenations, (iii) a bilinear layer was inserted in the middle of the EfficientNetV2 SE-block. A detailed study on this architecture is presented in (3).

BHI: Their approach adopts a “sandwich” architecture consisting of a one-dimensional convolutional layer, a bidirectional long-short-term memory (Bi-LSTM) layer, and another convolutional layer. Each convolutional layer used different kernel sizes. Besides the model architecture, the team found that training details specialized for DNA sequence-based deep learning models were highly important for the overall performance. Among them, the most crucial was to use a ‘post-hoc conjoined’ setting (4), which imposes a reverse-complement equivariance to the model. Test-time augmentation was also effective. Predictions were made for an original sequence, its four shifted variants (generated by -2bp, -1bp, +1bp, and +2bp shifting), and their reverse-complement sequences, then those 10 predictions were averaged to make a final prediction. While training sequences over 110bp were trimmed to the right, sequences shorter than 110bp were randomly padded with the original vector sequences on both sides. This informative padding gave a nonnegligible performance boost. Finally, to be as unbiased as possible for the distribution of the test set, predictions were quantile-transformed using the distribution of expression levels in training data as post-processing.

Unlock_DNA: The team used an end-to-end Macaron-like Transformer encoder architecture with two half-step feed-forward (FFN) layers at the beginning and end of each encoder block. A separable 1D convolution layer was inserted after the first FFN layer and in front of the multi-head attention layer. The sliding k-mers from one-hot encoded sequences were mapped onto a continuous embedding, combined with the learned positional embedding and strand embedding (forward strand vs. reverse complement strand) as the sequence input. Along with the sequence input, several positions (32 in the final model) of “pseudo” expression values were added as the input, where all input expression values were zeros. The model predicted one expression value

for each “pseudo” expression position and used the mean of the prediction of all positions as the final predicted expression value. A detailed study on this solution is presented on (5).

Camformers: This team used a CNN with residual connections. The model included six convolutional layers with three residual connections allowing the model to bypass every other layer. After the penultimate convolutional layer, a max pooling operation was added to reduce the model size and improve generalization. The output of the final convolutional layer was flattened and fed into a block of two dense layers, followed by a final dense layer outputting the predicted expression level. All layers except the last used a rectified linear unit activation.

NAD: The approach has two stages: (i) generating the embedding vectors for each base position using GloVe (6) and (ii) using the embedding vectors as input of neural networks to predict the gene expression level. The proposed model combines a convolutional neural network for feature extraction and a transformer for prediction.

WZTR: The team used a fully CNN-based architecture. The model begins with two convolutional layers, and six convolution blocks follow these layers. Each convolution block is constructed of 3 convolutional layers and an average pooling at the end. Each convolutional layer consists of a hybrid convolution (7), batch normalization, ReLU activation, and residual connection. Each hybrid convolution takes in a list of dilation values [1,2,4,6], with 4 convolutions processing the input in parallel. Finally, there are three fully connected layers and an output layer. A linear combination of 256 features extracted from all the previous operations on the sequence is used to generate the predicted expression.

High Schoolers Are All You Need (High Schoolers): This team used a mix of CNN and transformer architectures, where the CNN was based on Residualbind’s (8) design, with a convolutional layer (with exponential activations) followed by a residual block comprised of a series of dilated convolutional layers with increasing dilation rates. It was followed by attention pooling, a transformer layer with relative positional encodings, and a standard MLP block.

BioNML: The underlying neural network was configured to have a relatively larger set of convolutional kernels and extra dictionaries of short k-mers for spotting potential enriched DNA sequence patterns. Strand-specific streams of these patterns were normalized and consolidated with Swish activation’s (9) fully learnable thresholding. The encoded patterns were fed into a ViT (10) like block but with transformer decoder type of connections and SwiGLU (11) activations for modeling any sequential interdependence. A set of suppressed signals of the encoded sequence-based patterns as queries for the transformer decoder blocks to respond to.

BUGF: A transformer model was used to predict the expression bin classes, as opposed to treating the problem as a regression problem. Random mutations were added to the sequence as an data augmentation strategy and the model was trained to predict where the mutations had been made to the input sequence. An auxiliary loss was calculated based on this prediction, which helped reduce overfitting.

Mt: The approach uses GRU and CNNs to regress the strength of the targeted promoters using information encoded in the forward and reverse DNA strands.

SYSU-SAIL-2022: The team first trained a 3-layer BERT (12) using the top 20% of sequences in terms of expression. Then, the BERT embedding was used to train an expression predictor.

Wen Group: A deep neural network that adopted concepts of U-Net (13), Transformer, and Squeeze & Excitation blocks (14) was trained from end to end without any data augmentation.

Yuanfang Guan: A neural network that consisted of LSTM layers followed by attention layers was used to predict expression.

Metformin-121: A neural network based on bidirectional GRU was used to predict gene expression.

NGT4: A neural network based on XceptionNet (15) was used to predict gene expression. During training, the expression values were transformed evenly in the range of $\text{€} - 0.5 < x < \text{€} + 0.5$ (here, € is the integer expression) maintaining the ranking of sequences that was produced by a trained model.

Davuluri lab: The team utilized a transformer-based representation model named DNABERT (16) for predicting gene expression.

*DNABERT was pretrained on human genome, which violated the competition rules. However, we consider this to be an important benchmark that shows the limitation of DNA language models.

Wan&Barton_BBK: The team designed a model based on Temporal Convolutional Networks (17) to predict expression.

Peppa: The team designed a model based on the Enformer (18) that took 110 bp as input (compared to 200 kbp in the original) and included only 2M parameters (compared to ~200M parameters of the original).

The Dream Team: A neural network was used that incorporated convolutional, multihead attention, and LSTM layers. During training, the integer expression values were transformed by replacing them with Normal(€,0.3) distribution, where € represents the expression.

Noisy-Chardonnay: A model composed of convolutional layers followed by BiLSTM layers was trained without any data augmentation to predict expression levels.

KircherLab: The team trained a simple convolutional neural network with a GC correction step on the training data to help the model focus its decisions on motifs within the sequence rather than the general nucleotide composition.

MadLab: The model is composed of three building blocks, namely a convolutional network, a transformer and a recurrent network.

Auth: A simple hybrid architecture combining a convolutional layer and a BiLSTM layer followed by two fully connected layers was used to predict expression.

UTKbioinformatics: A neural network based on BERT was used to predict expression.

DrAshokAndFriends: An attention based ConvLSTM (19) model was used for prediction.

QUT_seq2exp: A sequence embedding model, dna2vec (20), was applied on the promoter sequences (in a running manner on short k-mers), which are then subsequently used as features for a transformer-based deep neural network model.

Zeta: A transformer model was used for predicting expression.

Supplementary Tables

Supplementary Table 1: Primers used in this study.

Name	Sequence (5'-3')	Orientation	Description
pT-pA_GibsRI	GAACTGCATTTTTTTCACATCNNN NNNNNNNNNNNNNNNNNNNNNNN NNNNNNNNNNNNNNNNNNNNNNN NNNNNNNNNNNNNNNNNNNNNNN NNNNNNNNNNNGGTTACGGCTGT TTCTTAA	Fw	Random promoter oligo for use in pTpA promoter context
R-pT_GibsDS	TTAAGAAACAGCCGTAACC	Rv	For doublestranding pTpA_GibsRI
Nextera_i5LN5_GpT	TCGTCGGCAGCGTCAGATGTGTA TAAGAGACAGNNNNNTGCATTTT TTTCACATC	Fw	Nextera adaptor addition, with 5 random bases to help clustering
Nextera_i7R_GpA	GTCTCGTGGGCTCGGAGATGTGT ATAAGAGACAGAACAGCCGTAAC C	Rv	Nextera adaptor addition

Supplementary Figures

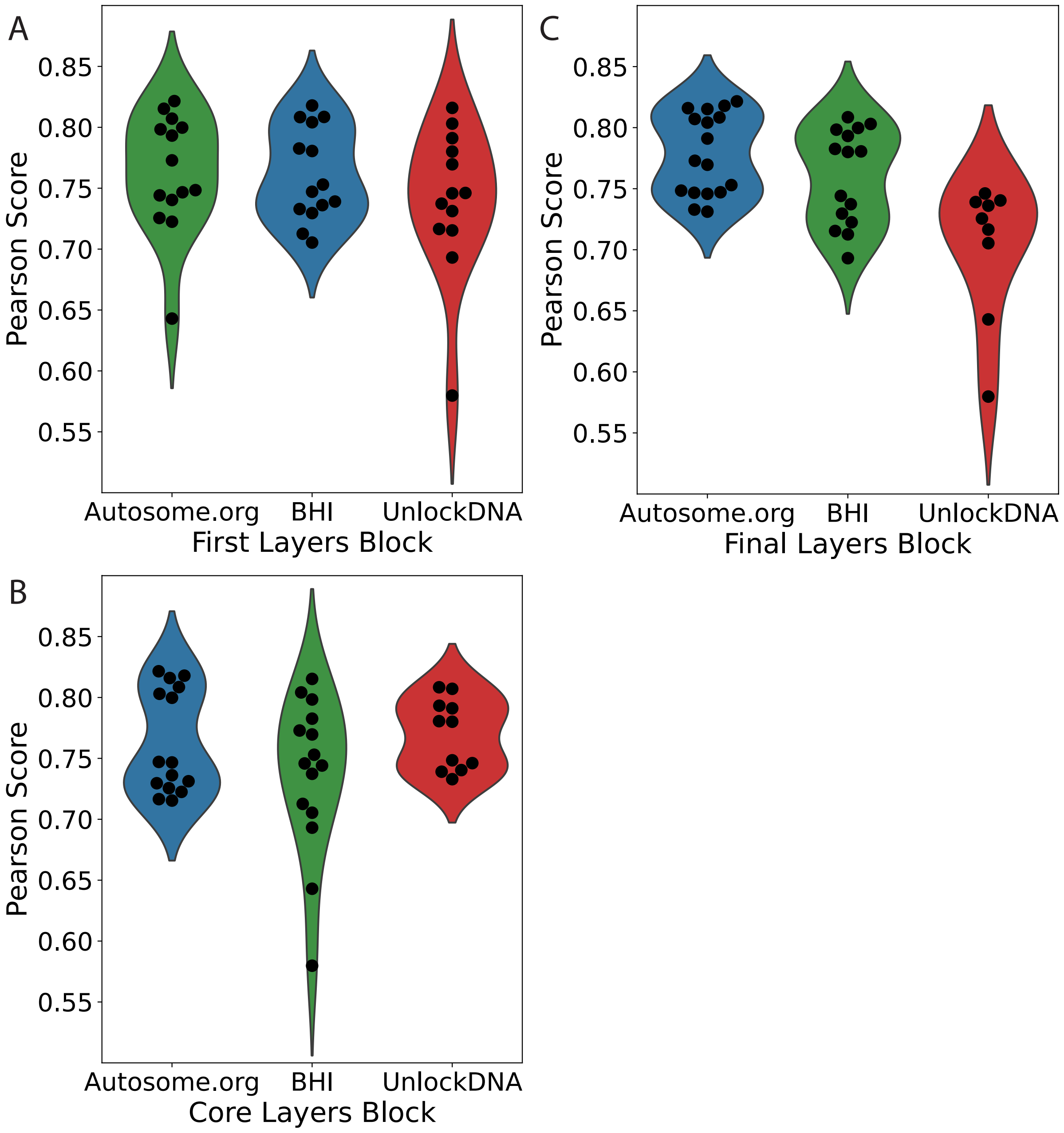

Supplementary Figure 1: No clear winners in other module blocks. Performance of the different teams' modules (*x*-axes and colours) in Pearson Score (*y*-axes) for the **(A)** First Layers, **(B)** Core Layers, and **(C)** Final Layers modules.

Supplementary References

1. Tan, M.; Le, Q. EfficientNetV2: Smaller Models and Faster Training. In *Proceedings of the 38th International Conference on Machine Learning*; PMLR, 2021; pp 10096–10106.
2. Krizhevsky, A.; Sutskever, I.; Hinton, G. E. ImageNet classification with deep convolutional neural networks. *Communications of the ACM* **2017**, *60*(6), 84–90. doi:10.1145/3065386.
3. Penzar, D.; Nogina, D.; Noskova, E.; Zinkevich, A.; Meshcheryakov, G.; Lando, A.; et al. LegNet: a best-in-class deep learning model for short DNA regulatory regions. *Bioinformatics* **2023**, *39*(8), btad457. doi:10.1093/bioinformatics/btad457.
4. Zhou, H.; Shrikumar, A.; Kundaje, A. Towards a Better Understanding of Reverse-Complement Equivariance for Deep Learning Models in Genomics. In *Proceedings of the 16th Machine Learning in Computational Biology meeting*; PMLR, 2022; pp 1–33.
5. Kwak, I.-Y.; Kim, B.-C.; Lee, J.; Garry, D. J.; Zhang, J.; Gong, W. Proformer: a hybrid macaron transformer model predicts expression values from promoter sequences. bioRxiv March 12, 2023, p 2023.03.10.532129. doi:10.1101/2023.03.10.532129.
6. Pennington, J.; Socher, R.; Manning, C. GloVe: Global Vectors for Word Representation. In *Proceedings of the 2014 Conference on Empirical Methods in Natural Language Processing (EMNLP)*; Association for Computational Linguistics: Doha, Qatar, 2014; pp 1532–1543. doi:10.3115/v1/D14-1162.
7. Yu, F.; Koltun, V. Multi-Scale Context Aggregation by Dilated Convolutions. arXiv April 30, 2016. doi:10.48550/arXiv.1511.07122.
8. Koo, P. K.; Majdandzic, A.; Ploenzke, M.; Anand, P.; Paul, S. B. Global importance analysis: An interpretability method to quantify importance of genomic features in deep neural networks. *PLOS Computational Biology* **2021**, *17*(5), e1008925. doi:10.1371/journal.pcbi.1008925.
9. Ramachandran, P.; Zoph, B.; Le, Q. V. Searching for Activation Functions. arXiv October 27, 2017. doi:10.48550/arXiv.1710.05941.
10. Dosovitskiy, A.; Beyer, L.; Kolesnikov, A.; Weissenborn, D.; Zhai, X.; Unterthiner, T.; et al. An Image is Worth 16x16 Words: Transformers for Image Recognition at Scale. arXiv June 3, 2021. doi:10.48550/arXiv.2010.11929.
11. Shazeer, N. GLU Variants Improve Transformer. arXiv February 12, 2020. doi:10.48550/arXiv.2002.05202.
12. Devlin, J.; Chang, M.-W.; Lee, K.; Toutanova, K. BERT: Pre-training of Deep Bidirectional Transformers for Language Understanding. arXiv May 24, 2019. doi:10.48550/arXiv.1810.04805.
13. Ronneberger, O.; Fischer, P.; Brox, T. *U-Net: Convolutional Networks for Biomedical Image Segmentation*. arXiv.org. <https://arxiv.org/abs/1505.04597v1> Accessed 23.04.19.
14. Hu, J.; Shen, L.; Albanie, S.; Sun, G.; Wu, E. Squeeze-and-Excitation Networks. arXiv May 16, 2019. doi:10.48550/arXiv.1709.01507.
15. Chollet, F. Xception: Deep Learning with Depthwise Separable Convolutions. arXiv April 4, 2017. doi:10.48550/arXiv.1610.02357.
16. Ji, Y.; Zhou, Z.; Liu, H.; Davuluri, R. V. DNABERT: pre-trained Bidirectional Encoder Representations from Transformers model for DNA-language in genome. *Bioinformatics* **2021**, *37*(15), 2112–2120. doi:10.1093/bioinformatics/btab083.
17. Lea, C.; Flynn, M. D.; Vidal, R.; Reiter, A.; Hager, G. D. Temporal Convolutional Networks for Action Segmentation and Detection. arXiv November 16, 2016. doi:10.48550/arXiv.1611.05267.
18. Avsec, Ž.; Agarwal, V.; Visentin, D.; Ledsam, J. R.; Grabska-Barwinska, A.; Taylor, K. R.; et al. Effective gene expression prediction from sequence by integrating long-range interactions. *Nature Methods* **2021**, *18*(10), 1196–1203. doi:10.1038/s41592-021-01252-x.
19. Shi, X.; Chen, Z.; Wang, H.; Yeung, D.-Y.; Wong, W.; Woo, W. Convolutional LSTM Network: A Machine Learning Approach for Precipitation Nowcasting. arXiv September 19, 2015. doi:10.48550/arXiv.1506.04214.

20. Ng, P. dna2vec: Consistent vector representations of variable-length k-mers. arXiv January 23, 2017. doi:10.48550/arXiv.1701.06279.
21. de Boer, C. G.; Vaishnav, E. D.; Sadeh, R.; Abeyta, E. L.; Friedman, N.; Regev, A. Deciphering eukaryotic gene-regulatory logic with 100 million random promoters. *Nature Biotechnology* **2020**, *38*(1), 56–65. doi:10.1038/s41587-019-0315-8.
